# Administration of ethiodized poppy seed oil-based contrast agent into the uterus enhances fertilization rate in mice inseminated with low sperm numbers

**DOI:** 10.1093/humrep/deaf204

**Published:** 2025-11-03

**Authors:** Edgar Del Llano, Marlene Rasschaert, Christophe Arnoult, Philippe Robert, Pierre F Ray, Corinne Loeuillet

**Affiliations:** Team Genetics Epigenetics and Therapies of Infertility (GETI), Institute for Advanced Biosciences, INSERM U 1209, CNRS UMR 5309, University Grenoble Alpes, Grenoble, France; Research and Innovation Department, Guerbet, Roissy CdG, France; Team Genetics Epigenetics and Therapies of Infertility (GETI), Institute for Advanced Biosciences, INSERM U 1209, CNRS UMR 5309, University Grenoble Alpes, Grenoble, France; Research and Innovation Department, Guerbet, Roissy CdG, France; Team Genetics Epigenetics and Therapies of Infertility (GETI), Institute for Advanced Biosciences, INSERM U 1209, CNRS UMR 5309, University Grenoble Alpes, Grenoble, France; UM GI-DPI, CHU Grenoble Alpes, Grenoble, France; Team Genetics Epigenetics and Therapies of Infertility (GETI), Institute for Advanced Biosciences, INSERM U 1209, CNRS UMR 5309, University Grenoble Alpes, Grenoble, France

**Keywords:** infertility, OSCM, Lipiodol^®^, sperm, insemination, hysterosalpingography, embryo

## Abstract

**STUDY QUESTION:**

Does the administration of an oil-based iodinated contrast medium (OSCM) in the mouse uterus have a positive effect on fertility similarly to what is observed in women following hysterosalpingography (HSG)?

**SUMMARY ANSWER:**

Following IUI of a small number of sperm (mimicking oligozoospermia), fertilization and birth rates were improved in female mice who had previously received an intrauterine administration of an OSCM confirming a pro-fertility effect.

**WHAT IS KNOWN ALREADY:**

HSG is a common diagnostic procedure used in infertile or subfertile women, primarily to assess the patency of fallopian tubes. It consists in injecting an iodinated contrast medium into the uterus to enable the imaging of the female reproductive tract by radiography. In the absence of obstruction, the contrast medium fills the uterus and transits through the fallopian tubes before overflowing in the peritoneal cavity. Several studies reported that in the few months following HSG, women injected with an OSCM (ethyl esters of iodinated fatty acids of poppy seed oil) had an enhanced pregnancy rate (either spontaneous or following IUI) compared to those administered with a water-based contrast medium. This suggests that OSCM administration in the female reproductive tract could improve fertility. However, the physiological mechanisms underlying this potential effect remain unknown.

**STUDY DESIGN, SIZE, DURATION:**

OSCM or PBS (phosphate buffered saline, control) were administered into the uteri of B6D2 female mice (5–9 weeks old, total n = 73). Two weeks later, intrauterine insemination was performed with mouse epididymis sperm from B6D2 male mice (8–16 weeks old). Females were euthanized and fertilized oocytes collected and incubated for 4 days up to the blastocyst stage (n = 52). Alternatively, females were left until delivery (n = 21).

**PARTICIPANTS/MATERIALS, SETTING, METHODS:**

Female nulliparous mice were administered either with PBS or OSCM directly into the uterus. After 2 weeks, females were inseminated with a controlled number of sperm. The resulting fertilized/unfertilized oocytes were collected and counted the following day to calculate the fertilization rates and then further incubated *in vitro* to follow the development to the blastocyst stage. In addition, females were mated with vasectomized males to allow implantation, subsequent pregnancy and birth.

**MAIN RESULTS AND THE ROLE OF CHANCE:**

First, females were inseminated with a high number of sperm (5 × 10e5 spermatozoa). Comparable fertilization rates (67.0 ± 5.4% and 60.0 ± 8.0%, *P* > 0.05) were observed from oocytes originating from OSCM and PBS-administered mice, respectively. This showed that when inseminating with a high number of sperm, uterine OSCM administration had no deleterious effect but did not improve fertilization. We postulated that the potential beneficial effect of OSCM administration might be masked by the optimal reproductive system of young mice. It was therefore decided to test the effect of OSCM in suboptimal reproductive conditions such as those observed when sperm concentration is low (oligozoospermia). As could be expected, fertilization rate decreased proportionally to the reduction of inseminated sperm suggesting that it is a relevant model for oligozoospermia and to mimic subfertility. When using a suboptimal number of spermatozoa (15 × 10e3), the fertilization rate was significantly increased in OSCM administered mice compared to controls (29.9 ± 4.8% and 12.6 ± 3.0%, *P* < 0.05, respectively). Moreover, OSCM administered females also gave birth to a higher number of pups than controls (3.7 ± 1.7 and 0.9 ± 0.9, *P* < 0.05).

**LARGE SCALE DATA:**

No large scale data were produced in this study.

**LIMITATIONS, REASONS FOR CAUTION:**

The reason for studying the effects of oil soluble iodinated contrast media on mouse fertility is based on the results from several clinical studies in humans. Those reports included women of different age and suffering from different types of infertility or subfertility. Moreover, the timing of their pregnancies after OSCM treatment also occurred at different times. Our mouse model and experiments do not include such heterogeneity as the inclusion of variables related to age, time of pregnancy and fertility deficiencies, which would demand a much larger number of mice, contrary to the 3R principles (Replacement, Reduction and Refinement of animal experimentation). For the same reason, our results are based upon mice treated with OSCM in contrast to mice treated with PBS, while human clinical reports may include alternatives to OSCM. Moreover, it is important to note that mouse and human reproductive systems differ at several levels such as morphology, hormonal influences, oestrus, menstrual cycles and pregnancy, raising the possibility that OSCM action may differ across species. In our mouse model, OSCM does not cross the uterotubal junction in the mice during administration, restricting it to the uterus, thus excluding any possible effects on the oviducts and peritoneal cavity, which are reached by OSCM in humans. Additionally, as the mouse reproductive cycle is much faster than that of women, it is likely that the amount of OSCM remaining in the mouse uterus after 2 weeks differs from what is left in women after several months.

**WIDER IMPLICATIONS OF THE FINDINGS:**

In our mouse model, OSCM administration in the uterus increased the number of embryos and pups obtained when inseminated with low sperm numbers. This suggests that the increased fertilization rate is most probably due to an effect on the uterus itself or on the sperm which go through it before reaching the fertilization site. In the mouse, the presence of OSCM in the oviduct and inside the peritoneal cavity is not necessary to observe a beneficial effect of the contrast agent. These initial results demonstrate that the mouse model could be used to further study the OSCM mechanisms in the uterus. Most importantly, our results confirm a beneficial effect of OSCM on fertility and suggest that it could help couples with fertility issues, improving fertilization and pregnancy rates following IUI with substandard sperm samples.

**STUDY FUNDING/COMPETING INTEREST(S):**

This work was supported by Guerbet Group, INSERM, CNRS, Université Grenoble Alpes and the French Agence Nationale pour la Recherche (ANR) grant “LIPIOFERT” (ANR-23-CE18-0039) to C.L. Guerbet Group is producer and distributor of Lipiodol^®^, provided by Guerbet free of charge for this study. The study was partly financed by Guerbet and M.R. and P.Ro. are Guerbet’s employees. Data were produced and analysed independently by academic researchers from the GETI’s laboratory E.L., C.A., P.Ra., and C.L. E.L. was temporarily employed by Guerbet from 1 December 2022 to 31 May 2023.

## Introduction

Infertility affects 50–70 million couples worldwide, which translates to 9–15% of couples who are actively seeking to have children (Thonneau *et al.*, 1991; Szamatowicz and Szamatowicz, 2020). This ailment is defined as the inability to conceive after 1 year of unprotected sexual intercourse (Zegers-Hochschild *et al.*, 2009). Infertility can be caused by numerous factors, affecting men, women or both (Thonneau *et al.*, 1991). Identifying the specific reasons behind each case is central in order to choose the right treatment to overcome infertility.

One of the prognosis techniques for infertility in women is hysterosalpingography (HSG), consisting of administering an iodinated contrast media into the female uterus which can be easily monitored through X-ray radiology (Schankath *et al.*, 2012). During the injection, the media fills the uterus completely and then flows through the fallopian tubes before coming out into the peritoneal cavity. This medical test therefore allows the detection of obstructions of the fallopian tubes and of potential uterine abnormalities (Zafarani *et al.*, 2021). Two types of contrast media are available for HSG: oil-soluble contrast media (OSCM), the most commonly used being Lipiodol^®^ which is composed principally of iodized poppyseed oil, and water-soluble contrast media (WSCM) with a wider number of products in use such as iobitridol (Xenetix^®^). Interestingly, several randomized controlled trials indicate that subfertile patients that underwent HSG had greater pregnancy rates (29–55%) compared to those who did not have any intervention (17%) (Mohiyiddeen *et al.*, 2015). Moreover, several clinical trials reported that HSG carried out with OSCM had a higher beneficial effect on infertility contrary to those performed with WSCM (Weir and Weir, 1951; Johnson, 2014). In a robust, multicentre, randomized controlled trial, it was reported that within 6 months of HSG, ongoing pregnancy rate for WSCM patients was 29.1% but reached 39.7% in the Lipiodol^®^ administered participants (Dreyer *et al.*, 2017). Moreover, in the same trial, live birth rates were also superior in the OSCM group (38.8%) compared to WSCM group (28.1%).

It is thus tempting to recommend HSG with OSCM as a method to alleviate unexplained infertility. Nonetheless, much research is still needed as the mechanisms by which Lipiodol^®^ enhances fertility remain unclear. Various theories have been proposed but with little evidence to support them. One of the first theories to emerge was the possibility that the pressure exerted by the contrast media administration into the uterus could remove potential debris blocking the fallopian tubes, thus making the passage for sperm more accessible after HSG (Wang *et al.*, 2019). However, it is not clear why OSCM could be more effective than WSCM at removing blocked tubes as both generate pressure in the tubes during the procedure. Another possibility would be that Lipiodol^®^ affects the intraperitoneal cavity and uterus, changing the composition and behaviour of dendritic cell populations and macrophages. Some studies showed that macrophages cultured in the presence of Lipiodol^®^ had their ability to phagocyte sperm inhibited, which would increase the chances of sperm survival and later oocyte fertilization (Johnson *et al.*, 1992; Mikulska *et al.*, 1994). In another study, Johnson *et al.* (2005) showed that intrauterine and intraperitoneal administration of Lipiodol^®^ in female mice significantly reduced the CD205^+^ dendritic cell population (responsible for overall antigen presentation) and increased the CD1^+^ population (responsible for presenting lipid antigens). It was postulated that these changes might alter the response of the immune system to the semi-allogenic conceptuses and limit their immune rejection. Alteration of immune cells has also been reported in women after HSG with Lipiodol^®^. Their peritoneal myeloid dendritic cells (MDCs) were more mature and less prone to phagocytosis and their regulatory T-cell (Treg) levels, which are crucial for feto-maternal tolerance, were higher (Izumi *et al.*, 2017). Moreover, the effect of OSCM, could be extended to the ovaries themselves as iodine (present in higher concentration in OSCM than in WSCM) is essential to produce thyroid hormone, which controls ovarian follicle growth and ovulation (Kobayashi *et al.*, 2009). Both insufficiency and overdose of iodine leads to deficient menstrual cycles and fertility problems in animals (Hidiroglou, 1979; Mahapatra and Chandra, 2017). It is therefore possible that the administration of OSCM helped subfertile patients reach optimal levels of iodine and subsequent better follicle growth and oocyte quality.

Research on this subject is not extensive and hard to pursue in humans. Reproducible data are easier to obtain using an animal model like the mouse, but there is a lack consensus on a specific protocol to administer Lipiodol^®^ on different animal models, and few studies have actually focused on the direct effect it might have on reproduction in mice (Johnson *et al.*, 2005; Mathews *et al.*, 2021).

For these reasons, in the present work we set to elucidate whether the reported effect of Lipiodol^®^ on fertility in women could be reproduced in a mouse model. Lipiodol^®^ was administered directly into the mouse uterus and, after a period of 2 weeks, intrauterine insemination was performed. After insemination with a reduced number of sperm, the number of embryos and delivered pups produced in Lipiodol^®^ administered mice were higher than in controls, thus confirming the effect of Lipiodol^®^ on mouse fertility.

## Materials and methods

### Ethics

Breeding and experimental procedures were carried out in accordance with national and international laws relating to laboratory animal welfare and experimentation (EEC Council Directive 2010/63/EU, September 2010). Experiments were performed under the supervision of C.L. (agreement 38 10 38) in the hTAG (Plateforme de Haute Technologie Animale Grenoble, France) animal care facility (agreement C3851610006 delivered by the Direction Départementale de la Protection des Populations) and were approved by the ethics committee of the PHTA and by the French government (APAFIS#35687-2022022411197687.v4 and APAFIS #13454-2018020814321031 v2). The mouse strain B6D2F1/JR was used for all experiments and was purchased from Janvier Laboratories (France). Supplementary Table S1 includes a detailed description of the number of mice used in each experiment and its replicates.

### Intrauterine administration

In order to synchronize oestrus cycle in B6D2 female mice, animals (between 5 and 9 weeks old) were hyperstimulated with injections of 5U of pregnant mare serum gonadotropin (PMSG) (Chronogest^®^ PMSG 600, MSD Santé Animale, France) followed by 5U of hCG (Chorulon^®^ 1500, MSD Sante Animale, France) 48 h later. Eleven hours after hCG injection, intrauterine administration of 50 µl of Lipiodol^®^ or PBS (control) was performed using a blunt syringe and the help of a plastic speculum to open the vagina.

### Visualization of Lipiodol^®^in the mouse reproductive tract

To check the correct administration of Lipiodol^®^ in the uterus, to determine the volume and to visualize its distribution afterwards, 5 mice were anesthetized with isoflurane (4% for induction and 2.5% for maintenance) (Zoetis, USA), then placed under X-ray radiography (Philips Veradius^®^) and Lipiodol^®^was gently administered until reflux.

Also, three mice were euthanized and their uteri exposed. Then a mix of Lipiodol^®^ and Brilliant Blue G dye (27815, Sigma, USA) (3 µg/ml) was administered via the cervix to visualize how the oil was delivered.

### Sperm collection and counting

B6D2 male mice (between 8 and 16 weeks old) were euthanized by cervical dislocation and their cauda epididymis were collected and placed in 1.5 ml of M2 medium (M7167, Sigma-Aldrich, USA) pre-warmed at 37 °C. Then, sperm was released into the medium by performing small incisions on the epididymis and left to swim for 10 min. The sperm quantity was then estimated using a Fast-read 102^®^ slide (Biosigma, Italy) after 1/100 sperm dilution in water.

### IUI, embryo collection and culture

For IUI, female B6D2 mice were hyperstimulated in the same way as for intrauterine administration. Then, depending on the experiment, the required number of spermatozoa was deposited into the uterus 11 h after hCG injection. Next morning (24 h later), female mice were euthanized, and their oviducts placed into pre-warmed M2 medium (M7167, Sigma-Aldrich, USA). Then, under a stereo microscope with a 37 °C heated plate, the oviducts were carefully dissected to allow the retrieval of the two-cell embryos as well as the non-fertilized oocytes. These were then transferred into KSOM medium (NaCl 95 mM (Euromedex, France), KCl 2.25 mM (VWR, USA), KH_2_PO4 367 µM (Sigma-Aldrich, USA), MgSO_4_.7H_2_O 203 µM (Sigma-Aldrich, USA), Glucose monohydrate 202 µM (Sigma-Aldrich, USA), Sodium lactate 1.67 mM (Sigma-Aldrich, USA), NaHCO_3_ 25 mM (VWR, France), Sodium pyruvate 182 µM (Sigma-Aldrich, USA), EDTA 13.7 µM (Sigma-Aldrich, USA), Glutamax™ 0.999 mM (Sigma-Aldrich, USA) supplemented with BSA 100 mg/ml, 1× NEAA (non-essential amino acids) (Gibco™, USA), 1× EAA (Essential amino acids) (Gibco™, USA), 1× penicillin/streptomycin (Sigma-Aldrich, USA) and CaCl_2_ 0.171 M (Sigma-Aldrich, USA) and covered with Mineral oil (Sigma-Aldrich, USA). They were further cultured at 37 °C and 5% CO_2_ in a humidified atmosphere to develop for 4 days. Each day, embryo development was observed under a stereo microscope.

In the experiment where females were expected to get pregnant and deliver pups, females were placed with vasectomized males for mating just after the IUI to stimulate uterus receptivity to embryos. After observation of a plug confirming mating, females were left until spontaneous delivery.

### Uterus histology

Two weeks after administration of PBS (control) or Lipiodol^®^, the uteri of two mice per condition were collected and fixed with 4% paraformaldehyde (PFA) (Electron Microscopy Sciences, USA) overnight at 4 °C. Tissues were then washed three times in PBS 1× followed by dehydration in a series of graded baths of 70%, 90%, 100% ethanol (Honeywell, USA) for 1 h each and a bath of xylene for 1 h (Carl Roth, Germany) before being embedded in paraffin (Leica Biosystems, Germany). Sections of 5 µm were cut with a microtome (Leica Biosystems, Germany) and placed onto Epredia™ Polysine Adhesive slides (Fischer scientific, USA). Paraffin was removed with xylene and then tissues were rehydrated in ethanol series (100%, 95%, 70%) before being stained for 15 s with Haematoxylin solution (Sigma Aldrich, USA) and counterstained for 2 min with Eosin phloxine B (Sigma Aldrich, USA). Tissue morphologies were observed with an AxioImager Z1, Zeiss microscope, oil objective ×63 (Zeiss, Germany).

### Statistical analysis

The data of this study are represented as the mean ± SEM of (n) individuals of at least three independent replicate experiments. The tests used to determine whether the differences between groups were statistically significant were Student’s t-test on the average or one-way ANOVA using the GraphPad Prism Software (San Diego, California, USA) with post-hoc analyses with a 95% confidence interval. * *P* < 0.05, ** *P* < 0.01, *** *P* < 0.001 considered as statistically significant.

## Results

### Establishment of a protocol to study the effect of Lipiodol^®^ in the mouse reproductive tract

**Figure 1.**
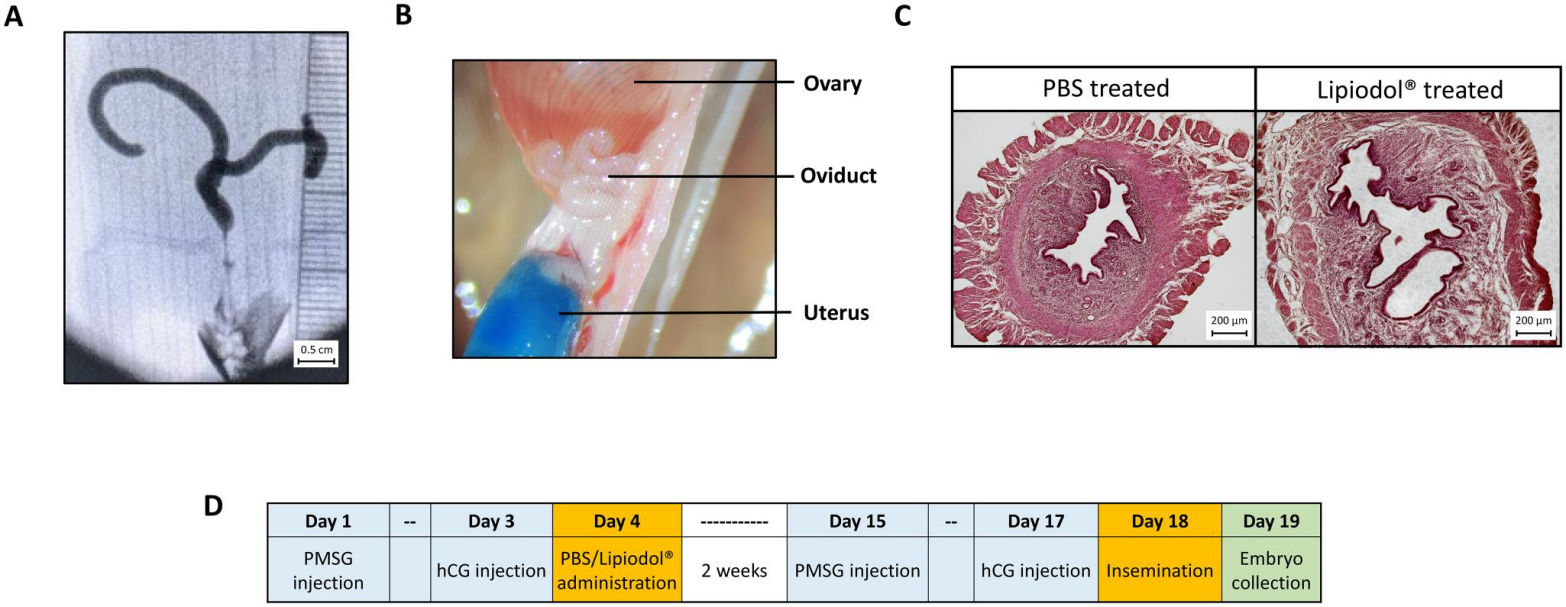
**Administration of Lipiodol^®^ into the mouse uterus**. (**A**) Visualization of Lipiodol^®^ contrast agent by using X-ray radiography. The agent completely fills the uterine horns. Scale bar 0.5 cm. (**B**) Picture of upper uterine horn, oviduct and ovary of a mouse administered with PBS (stained blue) shows that the liquid cannot cross the utertubal junction (UTJ) and does not enter the oviduct. (**C**) Histological uterine samples from mice administered with PBS (left) and Lipiodol^®^ (right) and stained with haematoxylin and eosin showing no differences in epithelium composition and integrity. Scale bar 200 µm. (**D**) Table depicting the protocol for the experimental test, including the injections of pregnant mare serum gonadotropin (PMSG) and hCG.

Our initial efforts aimed at adapting the HSG procedure to the mouse model. Using a blunt syringe (the same way as for IUI) to cross the cervix, we performed the administration of Lipiodol^®^ into the mouse uterus with different volumes of Lipiodol^®^ in female mice. Using X-ray radiography, we traced the way Lipiodol^®^ filled the female mouse reproductive tract (n = 5) (Fig. 1A). Similarly, we observed the path of dyed Lipiodol^®^ (after adding Brilliant Blue G dye to the product) after dissection of the female reproductive tract (n = 4) (Fig. 1B). We determined that 50 µl of Lipiodol^®^ filled the uterine cavity without overflowing back to the vagina. However, irrespective of the amount of Lipiodol^®^ injected, the oil could not cross the uterotubal junction (UTJ) during the injection and there was no trace of the contrast agent in the oviducts nor in the peritoneal cavity (Fig. 1A and B). This indicates that the mouse model would allow us to study the effects of Lipiodol^®^ on fertility at a compartmentalized level: the mouse uterine horns. We also performed histological analyses and noted that the Lipiodol^®^ treatment had no obvious deleterious effect on the uterine epithelium (n = 3 per experimental group) (Fig. 1C). No epithelial degeneration was detected as endometrial epithelial layers in the Lipiodol^®^ group showed no apparent changes at the level of tissue organization nor cell morphology.

We subsequently established a protocol to test if Lipiodol^®^ administration could affect mouse fertility in terms of fertilization rates or pre-implantation embryo development (Fig. 1D). First, mice were hormonally synchronized (with PMSG and hCG) and then Lipiodol^®^, or PBS as control, was administered through the cervix 11 h after hCG administration. Then, after a period of 14 days, the mice were again hormonally hyperstimulated followed by IUI of half a million sperm carried out 11 h after hCG administration. This 14-day period corresponded to 3 oestrus cycles in mice, which mimics the human 2–3 months delay reported to observe the pro-fertility action of Lipiodol^®^ in other studies. Finally, 24 h after IUI, two-cell embryos were collected and counted together with any other remaining unfertilized oocytes.

### Intrauterine administration of Lipiodol^®^ in mice does not alter their reproductive capacity and outcome

**Figure 2.**
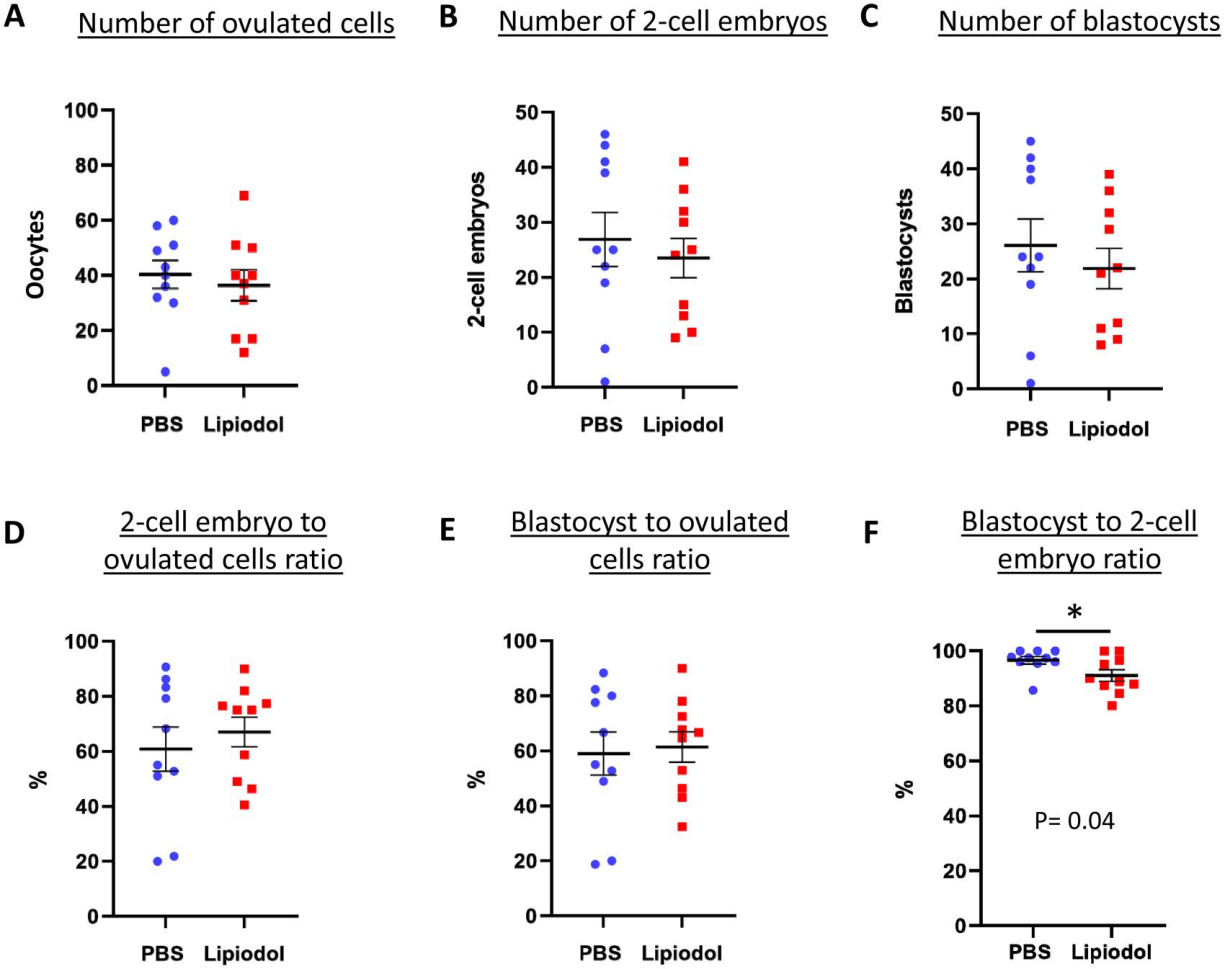
**Lipiodol^®^ uterine administration in mice has no effect on fertilization after intrauterine insemination (IUI) with 5 × 10e5 sperm**. The graphs show (**A**) the number oocytes ovulated, derived from the sum of unfertilized oocytes and embryos collected from each mouse oviducts 24 h after IUI, (**B**) the number of two-cell embryos collected and (**C**) the final number of blastocysts after culture, as well as (**D** and **E**) the ratio of two-cell embryos and blastocysts in relation to the total number of oocytes ovulated and (**F**) the developmental rate of two-cell embryos reaching the blastocysts stage. Data are presented as the mean ± SEM, and each dot represents an individual from three independent, replicate experiments (total n = 10 for each group), *P < 0.05 according to Student’s *t* test.

Our results did not report any significant difference in the number of cells ovulated between the two treatments (mean of 40.4 ± 5.0 for PBS (total n = 10) and 36.4 ± 5.6 for Lipiodol^®^ (total n = 10), Fig. 2A). The total number of two-cell embryos collected from the oviducts was also very similar with an average of 26.9 ± 4.9 and 23.5 ± 3.5 two-cell embryos in PBS- and Lipiodol^®^-administered mice, respectively (Fig. 2B). The total numbers of blastocysts were very similar to the number of two-cell embryos collected in both groups (26.1 ± 4.8 and 21.9 ± 3.7 blastocysts for PBS and Lipiodol^®^-administered mice respectively, Fig. 2C), indicating that most embryos developed correctly. Fertilization rates were not affected by Lipiodol^®^ treatment as the percentages of two-cell embryos and blastocysts in relation to the number of oocytes ovulated were very similar in PBS- and Lipiodol^®^- administered mice (mean of 60.8 ± 8.0% and 67.1 ± 5.3% of two-cell embryos; 59.1 ± 7.8% and 61.4 ± 5.5% of blastocysts, respectively, Fig. 2D and E). These results indicate that intrauterine administration of Lipiodol^®^ does not cause any detrimental effect on mouse fertility. Interestingly, a significant but only slightly superior blastocyst development rate was observed for the PBS compared to Lipiodol^®^-administered group (mean of 96.6 ± 1.3% and 91.1 ± 2.1%, *P* ≤ 0.05, Fig. 2F). This may indicate a minor decline in embryo quality caused by Lipiodol^®^ administration in mice but developmental rate still remained close to 100%.

### Development of an oligozoospermia-like phenotype by intrauterine insemination of a reduced number of spermatozoa

**Figure 3.**
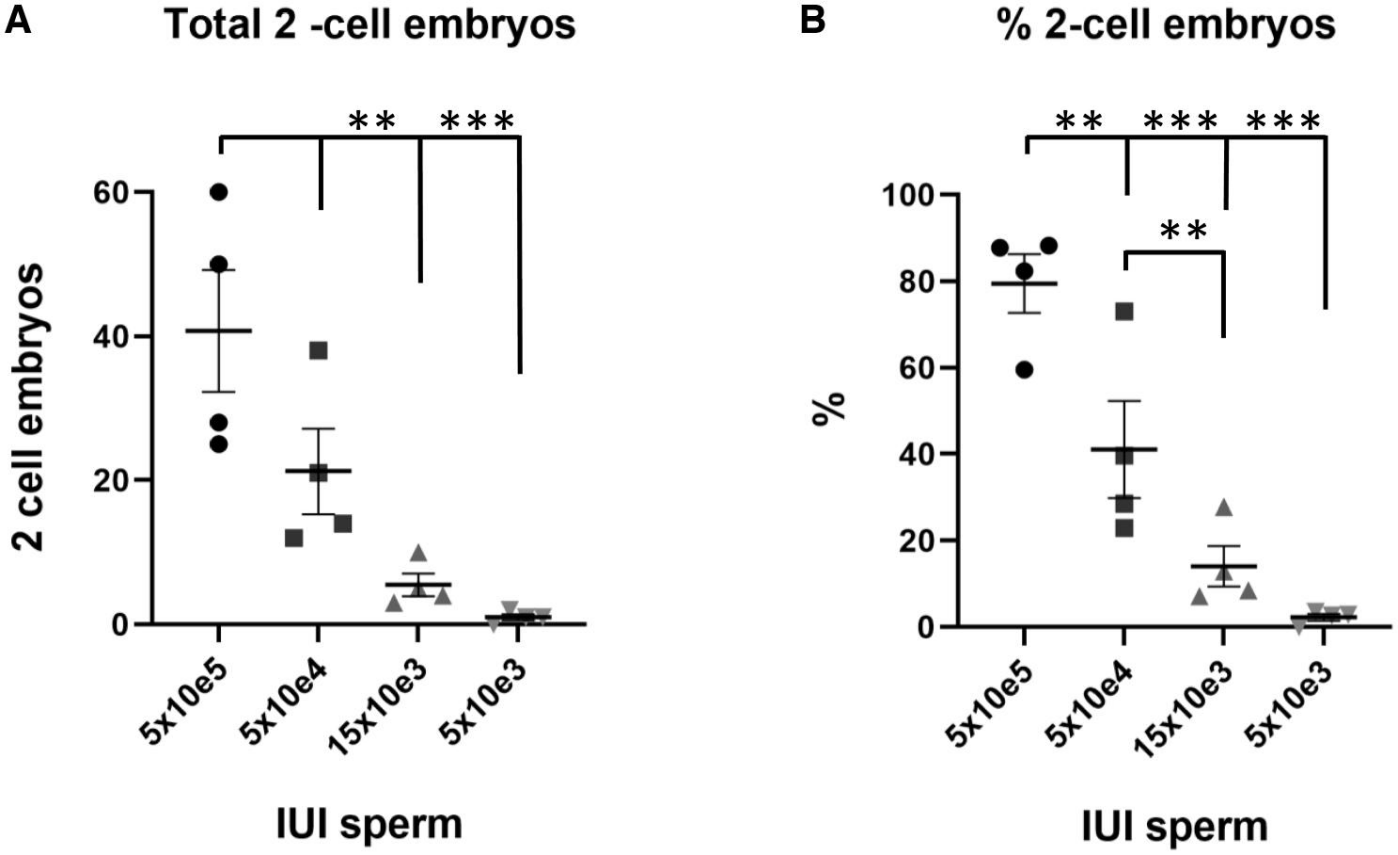
**Reducing the amount of sperm in IUI also decreases the number of fertilized oocytes in mice**. (**A**) The total number of collected two-cell embryos per mouse 24 h post-IUI is significantly reduced in mice inseminated with 15 × 10e3 sperm or less. (**B**) The ratio of two-cell embryos collected post-IUI is reduced when inseminated with 5 × 10e4, 15 × 10e3, and 5 × 10e3 sperm compared to 5 × 10e5 sperm. Data are presented as the mean ± SEM, and each dot represents an individual of four independent experiments (total n = 4 for each group), **P < 0.01, ***P < 0.001 according to one-way ANOVA test.

In the initial experiment, we demonstrated that Lipiodol^®^ had no deleterious effect on fertilization but we did not observe any improvement in fertility. Therefore, we hypothesised that the optimal fertility levels of young mice might be masking any potentially beneficial effects that Lipiodol^®^ administration might have. Moreover, all the studies reporting a Lipiodol^®^ pro-fertility effect were performed on couples experiencing some degree of infertility (Caglayan *et al.*, 2017; Dreyer *et al.*, 2017; Zhang *et al.*, 2022). To test this idea and to overcome the potential problem of mouse high fertility, we developed a mouse model with reduced fertility by inseminating female mice with decreasing amounts of sperm (from 5 × 10e5 to 5 × 10e3 sperm (total n = 4 per group)) followed by analysing the amount of *in vivo* fertilized two-cell embryos recovered and their subsequent preimplantation development. Specifically, we sought to find the right amount of sperm for IUI which would produce a small number of embryos without risking a total absence of fertilization. Interestingly, we observed an almost linear correlation between the number of inseminated sperm and the total number of two-cell embryos obtained (Fig. 3A). This correlation was also reflected at the level of two-cell embryo ratio in relation to the total number of oocytes ovulated (Fig. 3B). Inseminations with 5 × 10e5 and 5 × 10e4 sperm did not produce a significantly different number of embryos (mean of 40.0 ± 8.5 and 21.0 ± 5.9 two-cell embryos, respectively), while inseminating 15 × 10e3 and 5 × 10e3 sperm significantly decreased the number of obtained embryos (mean of 5.5 ± 1.5 and 1.0 ± 0.4 two-cell embryos respectively, *P* ≤ 0.05). A more consistent effect was observed when the number of obtained two-cell embryos were compared to the total amount of oocytes ovulated by each female (Fig. 3B). Each insemination with decreasing numbers of sperm produced a significantly smaller percentage of two-cell embryos (mean of 79.5 ± 6.7; 41.1 ± 11.2; 14.1 ± 4.7; 2.3 ± 0.8 for 5 × 10e5, 5 × 10e4, 15 × 10e3 and 5 × 10e3 sperm, respectively, *P* ≤ 0.05). Thus, we decided to use 15 × 10e3 sperm for the subsequent inseminations to reduce fertility, as inseminations with 5 × 10e3 sperm were too close to 0% fertilization rates and inseminations with 5 × 10e4 still produced a high number of two-cell embryos.

### Lipiodol^®^ administration enhances fertilization rates in females inseminated with limited sperm amounts

Having established that IUI with 15 × 10e3 sperm induced a reduced fertilization rate in female mice that could mimic the subfertility of the couples from Lipiodol^®^ trials, we proceeded to replicate our initial experiment with this limiting amount of sperm.

**Figure 4.**
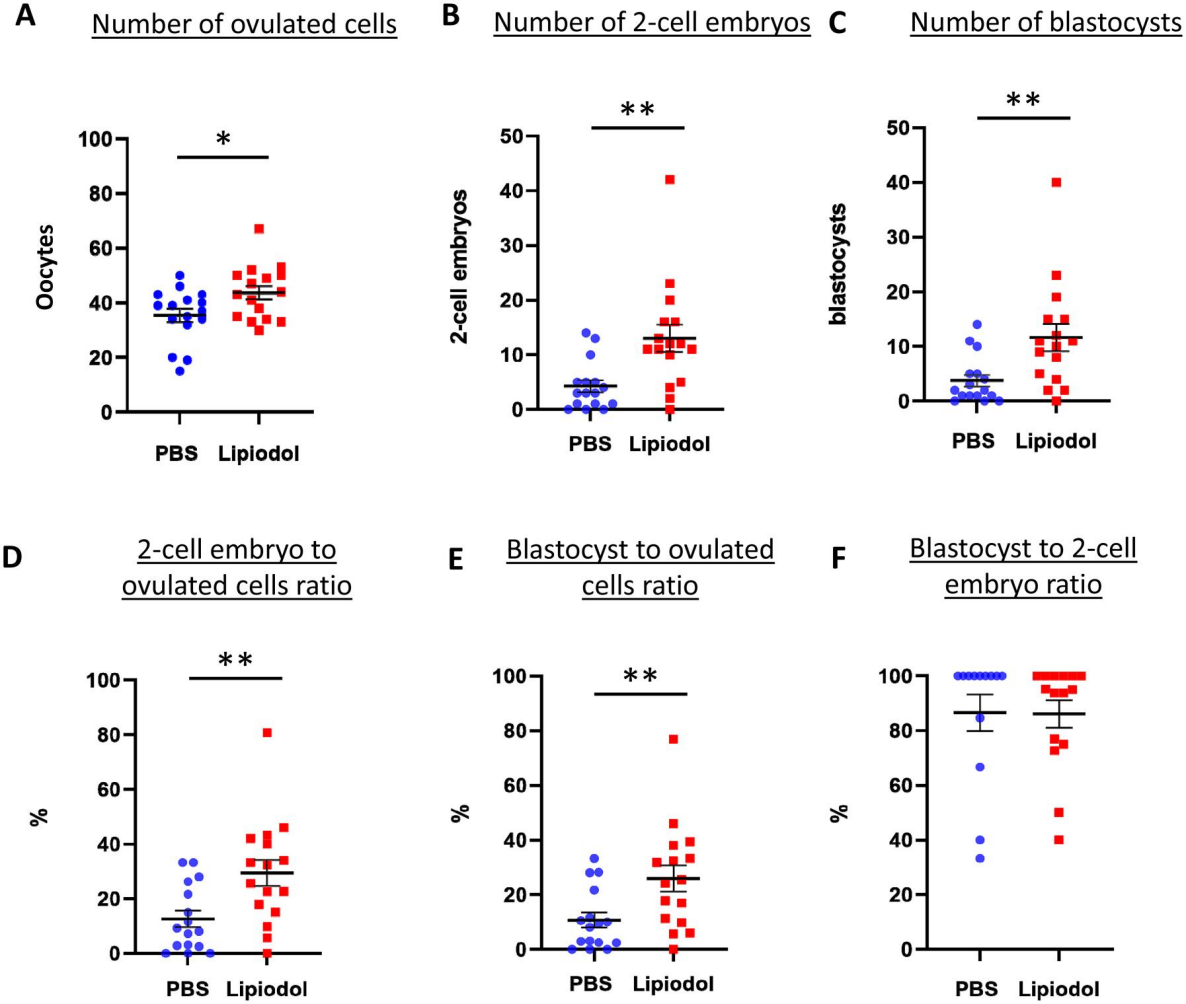
**Lipiodol^®^ uterine administration after IUI with reduced amounts of sperm (15**×**10e3 sperm) increases the number of fertilized oocytes**. The graphs show (**A**) a small increase of oocytes ovulated derived from the sum of unfertilized oocytes and embryos collected from each mouse oviducts 24 h after IUI, (**B**, **D**) Lipiodol^®^ treatment increases the number of two-cell embryos and its ratio (embryos/total oocytes ovulated), (**C**, **E**) Lipiodol^®^ increases the blastocyst amount and ratio (blastocysts/total oocytes ovulated) whereas the development from two-cell embryos to blastocyst is not affected (**F**). Data are presented as the mean ± SEM (standard error of the mean), and each dot represents an individual from three independent, replicate experiments (total n= 16 for each group), *P < 0.05, **P < 0.01 according to Student’s *t* test.

Interestingly, several of the parameters previously analysed presented a significant difference between PBS and Lipiodol^®^. The total number of oocytes (unfertilized and fertilized) collected was slightly but significantly increased after Lipiodol^®^ administration by 1.2-fold (with a mean of 35.5 ± 2.5 and 43.7 ± 2.5 for PBS (total n = 16) and Lipiodol^®^ (total n = 16) administered mice, respectively, *P* ≤ 0.05, Fig. 4A). More striking was the difference in the number of two-cell embryos obtained with an increase of 3-fold for Lipiodol^®^-administered females (4.3 ± 1.1 and 13.0 ± 2.5 for PBS and Lipiodol^®^, respectively, *P* ≤ 0.005, Fig. 4B). The production of two-cell embryos relative to the total number of ovulated oocytes was significantly increased by 2.3-fold (with a mean of 12.7 ± 3.0% and 29.5 ± 4.9% for PBS- and Lipiodol^®^-administered females, respectively, *P* ≤ 0.05, Fig. 4C). These results demonstrate an improvement of fertilization rates in the female mice administered with Lipiodol^®^ as opposed to PBS when inseminated with very low levels of sperm. We also looked for embryo development and found that the number of blastocysts (Fig. 4D) and the percentage of total oocytes reaching the blastocysts stage (Fig. 4E) were also increased by 3.1-fold and 2.4-fold in the Lipiodol^®^-administered mice when compared to the PBS-administered mice. Finally, the two-cell to blastocyst percentage, reflecting the number of two-cell embryos which reached the blastocyst stage, did not show any difference (mean of 86.5 ± 6.5 and 86.2 ± 5.0 for PBS- and Lipiodol^®^-administered, respectively, Fig. 4F) demonstrating that the increased blastocyst ratio (in relation to the total number of ovulated oocytes) of the administration group is due only to an improved fertilization in Lipiodol^®^-administered mice.

**Figure 5.**
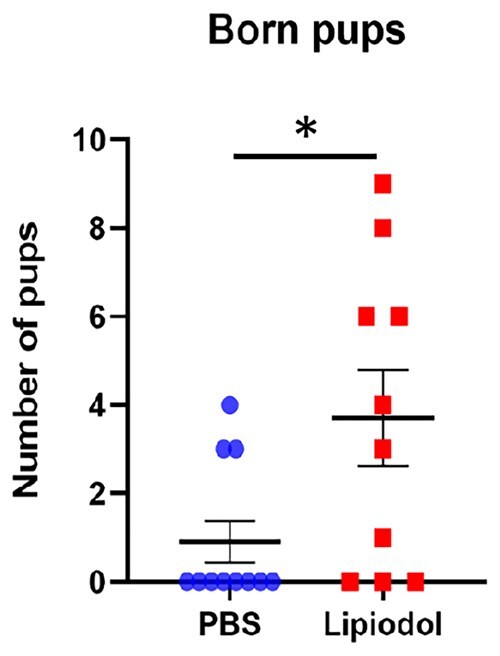
**Female mice administered with Lipiodol^®^ and IUI with reduced amounts of sperm (15**×**10e3 sperm) deliver a higher number of pups**. The graph shows an increased number of pups delivered after IUI in the Lipiodol^®^-administered group of females. Data are presented as the mean ± SEM (standard error of the mean), and each dot represents the number of pups delivered per individual from four independent, replicate experiments (total n = 11 and 10 for PBS and Lipiodol groups respectively), *P < 0.05, according to Student’s *t* test.

Furthermore, the effect of Lipiodol^®^ on spontaneous delivery was also analysed. Females were administered with PBS (total n = 11) or Lipiodol^®^ (total n = 10) exactly as in previous experiments, 2 weeks later they were inseminated with 15 × 10^3^ sperm, subsequently mated with vasectomized males and left to gestate until they gave birth. As expected from the small number of injected sperm, both PBS- and Lipiodol^®^- administered females had rather small litters (mean of 0.9 ± 0.8 pups and 3.7 ± 1.7 pups respectively, Fig. 5). However, Lipiodol^®^-administered females had an average litter size 4.1-fold larger than the PBS group (*P* ≤ 0.05). These results are concordant with the increase in fertilized oocytes observed in Lipiodol^®^-administered females, which would logically lead to an increase in implanted embryos and the subsequent number of pups born.

## Discussion

Our research presents a mouse model that reflects a positive effect of Lipiodol^®^ uterine administration on fertility. On one side, we have shown that in mouse with normal fertility conditions Lipiodol^®^ has no impact on embryo fertilization, either positive or negative. On the other hand, when fertility was hindered by inseminating with a reduced number of sperm, female mice administered with Lipiodol^®^ showed a significant increase in the number of two-cell embryos and delivered pups compared to the control, suggesting a positive effect of Lipiodol^®^ in fertilization. Moreover, we observed in this model that Lipiodol^®^ remained in the uterus and could not penetrate inside the oviduct. Together these results show that Lipiodol^®^ has a pro-fertility effect by either acting on the uterus or on the sperm, ultimately allowing the sperm to fertilize more oocytes. As such, this model could be used to further study this effect and help elucidate its mechanisms of action and translate them into new therapies for couples suffering from infertility.

In the last three decades, there has been growing evidence that couples suffering from unexplained infertility and who underwent HSG with OSCM achieved higher pregnancy rates compared to those with no treatment or HSG with WSCM (Weir and Weir, 1951; Johnson, 2014; Mohiyiddeen *et al.*, 2015; Dreyer *et al.*, 2017). However, the mechanisms driving the pro-fertility effects of OSCM have not been unravelled mainly due to (1) the limitations associated with research on human fertilization and (2) the difficulty of obtaining meaningful results due to the great genetic and phenotypic variability in patients. For that reason, we decided to test whether Lipiodol^®^ could improve fertility in the mouse and provide a model with less variability and more amenable to be used to study the physiological effect of Lipiodol^®^.

Initially, we adapted the HSG procedure to the mouse model. Lipiodol^®^ administration in the mouse was relatively simple by using a blunt syringe to gently cross the cervix (the same way as in IUI). In our protocol, we tested a 2-week period between the Lipiodol^®^’s administration and the IUI. This interval was chosen to mimic the span in humans between the HSG intervention and the first pregnancy reports (Caglayan *et al.*, 2017; Dreyer *et al.*, 2017; Zhang *et al.*, 2022). In most studies, the increased fertility rates observed after HSG treatment were reported between the second and sixth month, although some cases were recorded after up to 1 year. Therefore, the choice of a 2-week period in our protocol for mice (3 oestrus cycles) seemed appropriate although it might not necessarily be the best time frame to observe the maximal Lipiodol^®^ effect. To elucidate that, it would be necessary to perform the same experiments presented here with a total of 3, 4 or more weeks between HSG and IUI to examine if fertilization could be further improved.

Another feature to consider from the Lipiodol^®^ administration protocol presented here is the impossibility for the oil to cross the UTJ during the injection in this model while in women the contrast agent fills the uterus and crosses the fallopian tubes to flow into the peritoneal cavity. In mice, the oil was evenly distributed along the two uterine horns but could not enter the oviducts. On one hand, this prevented us from testing the effects Lipiodol^®^ could have on the oviducts and peritoneal cavity; on the other hand, this brings the opportunity to study the effects that Lipiodol^®^ might exert specifically in the uterus, pinpointing the observed effects to this specific organ. Therefore, the effects of Lipiodol^®^ on fertility we report on mice are most probably due either to changes in the uterus or to effects on the sperm deposited in the organ during inseminations. We, however, cannot exclude a limited diffusion through the UTJ or through the endometrium to the peritoneal cavity. We observed that the administration of Lipiodol^®^ in the mouse uterus did not induce any obvious damages to the uterus morphology as assessed by histological examination. This positive outcome is important, as large volumes of iodine administered into the uterus have been reported to cause oedema and haemorrhages in the lamina propria together with vacuolization and necrosis in mares (van Dyk and Lange, 1986). Also, the feeding of excessive doses of iodine has been described to be deleterious to rat ovaries and uterus (Mahapatra and Chandra, 2017; Mathews *et al.*, 2021).

In addition, it appeared that Lipiodol^®^ is not harmful to the sperm as our first results after IUI with a high number of spermatozoa did not show any difference in oocyte fertilization between control PBS and Lipiodol^®^ administrations. Interestingly, we detected a small but significant decrease in embryo development after Lipiodol^®^ administration in the experiments we performed with high sperm number IUI (5 × 10e5). However, this decrease was not observed on inseminations with low sperm number (15 × 10e3). This inconsistency between the two experiments suggests that Lipiodol^®^ is not directly responsible for a decrease in embryo development. Our experiments with low sperm IUI also reported a significant increase in the number of oocytes ovulated in the females administered with Lipiodol^®^. However, this difference from the control was not observed in the experiments with high sperm IUI. Thus, although we could not find a reason for such divergence, the cause most probably is not linked to any Lipiodol^®^ effect as the same amount of Lipiodol^®^ was administered in both low and high sperm IUI experiments.

To overcome the naturally high fertility of mice, we introduced a fertility handicap by reducing the number of sperm used for insemination. Interestingly, in this case, intra-uterine administration of Lipiodol^®^ led to an increase in both fertilized oocytes and delivered pups thus highlighting a positive effect of Lipiodol^®^ on fertilization rates. These findings contribute to the understanding of the mechanisms behind the Lipiodol^®^ pro-fertility effect and provide some elements to support or invalidate different hypotheses that have been suggested to explain it. For example, some suggest that the positive effects of HSG on couples with unexplained infertility might be due to the dislodging of debris allocated in the oviducts, thus opening the passage for sperm and oocytes alike (Wang *et al.*, 2019). However, this theory would not explain why HSG with OSCM has a stronger beneficial effect on fertility than with WSCM, given that both could dislodge oviductal plugs (Weir and Weir, 1951; Johnson, 2014). Moreover, our experiments demonstrate that neither PBS (control) or Lipiodol^®^ crosses from the uterus to the oviducts, likely due to the tight UTJ of mice. Although these results do not directly exclude the possibility of oviductal debris removal following human Lipiodol^®^ HSG, they more likely point towards an effect on the uterus and/or on the sperm present in it. That could also explain why HSG with OSCM results in better pregnancy rates compared to WSCM as both could be capable of debris removal but Lipiodol^®^ would have an additional effect on oocyte fertilization. Our findings are coherent with those of Mikulska *et al.* (1994), Johnson *et al.* (1992) and Izumi *et al.* (2017) who described that Lipiodol^®^ administration changed the immunological status of the uterus. These studies suggested that the changes in the immune environment would lead to a better survival of sperm to macrophages and also make the uterus more receptive to embryo implantation. However, while our results would support an increased sperm survival after Lipiodol^®^ administration, they do not directly relate to the implantation factor. In our results, we observed an increase in pups born in the Lipiodol^®^ group but, based on the current data, we cannot determine whether this is due to an improved implantation or not. The reported increase in delivered pups is definitely downstream of the higher fertilization rate observed, reinforcing Lipiodol^®^’s beneficial role in fertility. However, a possible effect on implantation cannot be excluded, and further experiments involving embryo implantation would be needed to verify this hypothesis.

Instead, as stated previously, our results point towards an indirect effect on sperm through the uterus; however, we cannot exclude a potential direct effect on the sperm itself. Lipiodol^®^ is known for its long half-life and long retention in the woman’s pelvis, up to 50 days after HSG (Miyamoto *et al.*, 1995). It is possible that Lipiodol^®^ molecules change sperm physiology in such a way that it increases their survival in the uterus or their ability to fertilize the oocyte. Afterall, lipids of the sperm plasma membrane play a crucial role in the sperm maturation process (Gautier and Aurich, 2022). For example, in order preserve sperm fertility after freezing, different media have been developed and improved by the addition of certain lipids (Spizziri *et al.*, 2010; Serin *et al.*, 2011; Tomás *et al.*, 2011). In the case of Lipiodol^®^, its contact with the sperm plasma membrane could influence its vitality or the pathways leading to capacitation and/or acrosome reaction in such a way that leads to increased oocyte fertilization.

In conclusion, we have developed a mouse model which reflects a pro-fertility effect of Lipiodol^®^ when directly administered into the uterus in an HSG fashion leading to an increase of fertilized oocytes and number of pups born. These outcomes following Lipiodol^®^ administration reveal that the effect is most probably taking place in the uterus and might impact the spermatozoa. Our mouse model represents a new tool to study the effects of Lipiodol^®^ in a controlled and reproducible manner while introducing new views about its relationship with fertility.

## Supplementary Material

deaf204_Supplementary_Table_S1

## Data Availability

All data underlying this article are available in the article and in its online supplementary material. No other or new data were generated or analysed in support of this research.
