# Supplementary material for: Administration of ethiodized poppy seed oil-based contrast agent into the uterus enhances fertilization rate in mice inseminated with low sperm numbers
Source: Hum Reprod. 2025 Nov 3;41(1):69–77. doi: 10.1093/humrep/deaf204 (PMC12769443; doi:10.1093/humrep/deaf204)
Supplement: deaf204_Supplementary_Table_S1 [file deaf204_supplementary_table_s1.pdf]

OXFORD
human reproduction
Human Reproduction, 2025, 1–1
https://doi.org/10.1093/humrep/deaf204
Supplementary Data

Supplementary Table S1. Number of mice used in each set of experiments and its replicates as seen in each figure.

**Figure 1**	A)	B)	C)		
PBS	–	1	3		
Lipiodol®	5	3	3		
**Figure 2**	Replicate 1	Replicate 2	Replicate 3	Total	
PBS	5	3	2	10	
Lipiodol®	5	2	3	10	
**Figure 3**	Replicate 1	Replicate 2	Replicate 3	Replicate 4	Total
5 × 10e5	1	1	1	1	4
5 × 10e4	1	1	1	1	4
15 × 10e3	1	1	1	1	4
5 × 10e3	1	1	1	1	4
**Figure 4**	Replicate 1	Replicate 2	Replicate 3	Total	
PBS	5	5	6	16	
Lipiodol®	5	6	5	16	
**Figure 5**	Replicate 1	Replicate 2	Replicate 3	Replicate 4	Total
PBS	3	3	2	3	11
Lipiodol®	3	2	2	3	10

© The Author(s) 2025. Published by Oxford University Press on behalf of European Society of Human Reproduction and Embryology.
This is an Open Access article distributed under the terms of the Creative Commons Attribution License (https://creativecommons.org/licenses/by/4.0/), which permits unrestricted reuse, distribution, and reproduction in any medium, provided the original work is properly cited.
Human Reproduction, 2025, 00, 1–9
https://doi.org/10.1093/humrep/deaf204
Original Article
